# Clinical presentation, management strategies, and mortality outcomes in idiopathic aortitis: a systematic review and meta-analysis

**DOI:** 10.1186/s43044-026-00795-8

**Published:** 2026-11-09

**Authors:** Omar Hamdan, Sarah Otoom, Noureddin Abdaljaleel, Ahmad Sandouka, Hams Alsakarneh, Nour Al-Ruwaidan, Sara Qreini, Aktham El Hadid, Hala Rabi, Francisco Arias

**Affiliations:** 1 University of Jordan, Amman, Jordan; 2 Jordan University of Science and Technology, Irbid, Jordan; 3 The University of Texas Rio Grande Valley, Edinburg, USA

**Keywords:** Aortitis, Idiopathic aortitis, Chest pain, Aneurysm, Isolated aortitis, Clinically isolated aortitis

## Abstract

**Introduction:**

Idiopathic Aortitis (IA) is defined as inflammation of the aortic wall, in the absence of a systemic disease or an infection. Most cases are found incidentally during repair of thoracic aortic aneurysms. Given the rarity of the disease, data on IA diagnosis and treatment are limited and derived mainly from small observational studies. This study aims to comprehensive and up-to-date synthesis of the available evidence on IA.

**Methods:**

Literature search of the PubMed, Scopus, and Web of Science databases was performed in late September 2025. Data regarding patients’ medical history, presentations, complications, diagnostic investigations, management and outcomes were obtained.

**Results:**

A total of 58 studies were included, comprising data from 79 patients with idiopathic aortitis. The mean age was 46 ± 20 years, with a slight male predominance of 53%. Weight loss was the most frequently reported symptom (58%). Regarding management, 44% underwent exclusive surgical treatment, 31% received mixed therapy, and 25% were managed medically. Corticosteroids were utilized in 51% of cases. The overall mortality rate was 20%. Significant predictors of mortality included a younger age at presentation (18 vs. 48 years, *P* < 0.001) and descending aorta involvement (*P* = 0.005). Conversely, corticosteroid administration (*P* = 0.002) and clinical surveillance (*P* < 0.001) were significantly associated with survival.

**Conclusion:**

This meta-analysis highlights idiopathic aortitis as a severe condition with significant mortality whereas the administration of corticosteroids and clinical surveillance are strongly associated with survival, our findings emphasize the need for standardized, evidence-based management protocols and prospective multicenter studies to optimize patient outcomes.

## Introduction

Aortitis is defined as inflammation of the aortic wall, and it has various etiologies. It is broadly divided into infectious type, mainly due to bacterial or fungal pathogens and noninfectious type, which most commonly involves large-vessel vasculitides like giant cell arteritis, Takayasu arteritis as well as IgG4-related disease [1–3]. In the absence of a systemic disease or an infection, this entity is designated Idiopathic Aortitis (IA) [4]. Although infrequent, IA is now more well-known in surgical and imaging series, with consequent clinical relevance [5].

The actual prevalence of IA is unknown, in part because of the absence of a well-established definition and its underdiagnosis [4]. The majority of cases are found incidentally during repair of thoracic aortic aneurysms; however, some are recognized by cross-sectional imaging for unrelated symptoms [5, 6]. When clinical symptoms occur, they tend to be nonspecific and systemic, comprising fever, malaise or polymyalgic signs that are similar to GCA or other vasculitic disorders [5, 7]. Diagnosis is mainly based on imaging. Computed Tomography (CT) scan and Magnetic Resonance Angiography (MRA) generally show circumferential/aortic wall thickening or aneurysmal dilatation, Positron Emission Tomography – Computed Tomography (PET-CT) can identify metabolically active, inflammatory, disease and assess disease extent [8, 9]. Despite these advancements, differentiating between IA and early or limited GCA or TAK continues to pose a challenge, especially in the absence of biopsy [7]. Histopathology typically demonstrates lymphoplasmacytic or granulomatous inflammation of the media and adventitia, occasionally with giant cells, but in the absence of systemic features fulfilling criteria for recognized forms of vasculitis [5, 7].

There is no established treatment protocol for IA treatment, and no widely accepted guidelines are available [7]. Glucocorticoids are often the first-line therapy, similar to other large-vessel vasculitides [3, 7]. On the other hand, some physicians choose to delay medical therapy until after the surgical repair of the aorta [7, 9].

Given the rarity of the disease, data on IA diagnosis and treatment are limited and derived mainly from small observational studies, mainly in the form of case reports and case series. This systematic review and meta-analysis aims to be the first study to provide a comprehensive and up-to-date synthesis of the available small observational studies on the demographic, clinical, laboratory, and imaging characteristics, and the management strategies and predictors of the outcomes of IA.

## Methods

### Literature search and protocol registration

An inclusive literature search of the PubMed, Scopus, and Web of Science databases was performed in late September 2025 using the keyword “idiopathic aortitis”, “Isolated Aortitis”, and “Clinically Isolated Aortitis” with no restrictions on language or publication date. The study adhered to the Preferred Reporting Items for Systematic Reviews and Meta-analysis (PRISMA) guidelines [10]. The study protocol was registered on the PROSPERO International prospective register for systematic reviews.

### Study selection and inclusion criteria

All retrieved articles were uploaded to the Rayyan web-based platform [11] to facilitate screening according to the predefined eligibility criteria. The inclusion criteria encompassed studies reporting patients diagnosed with “idiopathic aortitis” which was either histologically proven or a combination of clinical and radiological evidence with the exclusion of the other causes of the disease. Eligible study designs included case reports, case series, editorial reports describing cases of idiopathic aortitis, published in any language and at any time.

Exclusion criteria included non–peer-reviewed articles, newspapers, opinion pieces, review articles, conference abstracts or proceedings, book chapters, and animal studies. Studies with substantial missing or insufficient patient-level data were also excluded.

Each study was independently screened by two reviewers. In cases of disagreement between reviewers, the study was discussed with the senior investigator (OH) to reach a consensus. All discrepancies were resolved through discussion.

### Data extraction

A standardized spreadsheet was developed to extract relevant data from the included studies. Extracted variables comprised patient demographics (number of patients, age, and sex), medical history and comorbidities (including autoimmune diseases, diabetes mellitus, hypertension, malignancy, prior catheterization or stenting, and cardiovascular or cerebrovascular disease), and clinical presentation (chest, back, or abdominal pain; fever; fatigue; malaise; weight loss; night sweats; and claudication). Data on disease-related complications were also collected, including aortic dissection, rupture, aortic regurgitation, and aortic stenosis. Laboratory findings extracted included antinuclear antibody (ANA) status, C-reactive protein (CRP), and erythrocyte sedimentation rate (ESR), when available. Diagnostic evaluation variables included imaging modalities performed (ultrasound, X-ray, computed tomography, and magnetic resonance imaging) and their reported findings, as well as the availability and results of histopathological examination, detailing the pattern of aortic wall involvement and specific pathological features. Information regarding the initial and final diagnosis was collected when reported.

### Statistical analysis

Data analysis was performed RStudio (R version 4.4.1). Descriptive data, including patient demographics, medical history, comorbidities, clinical presentation, disease-related complications, laboratory findings, diagnostic evaluation, management strategies and outcomes, were reported as frequencies and percentages for categorical variables, whereas continuous variables were reported as means with standard deviations (± SD) or medians with ranges, as appropriate. GraphPad Prism version 8.0 was used to visualize the data and generate figures. Comparisons between disease outcomes among patients were performed using the Chi-square test, Fisher’s exact test, or independent samples t-test, as appropriate. A p-value of less than 0.05 was considered statistically significant.

## Results

**Fig. 1:**
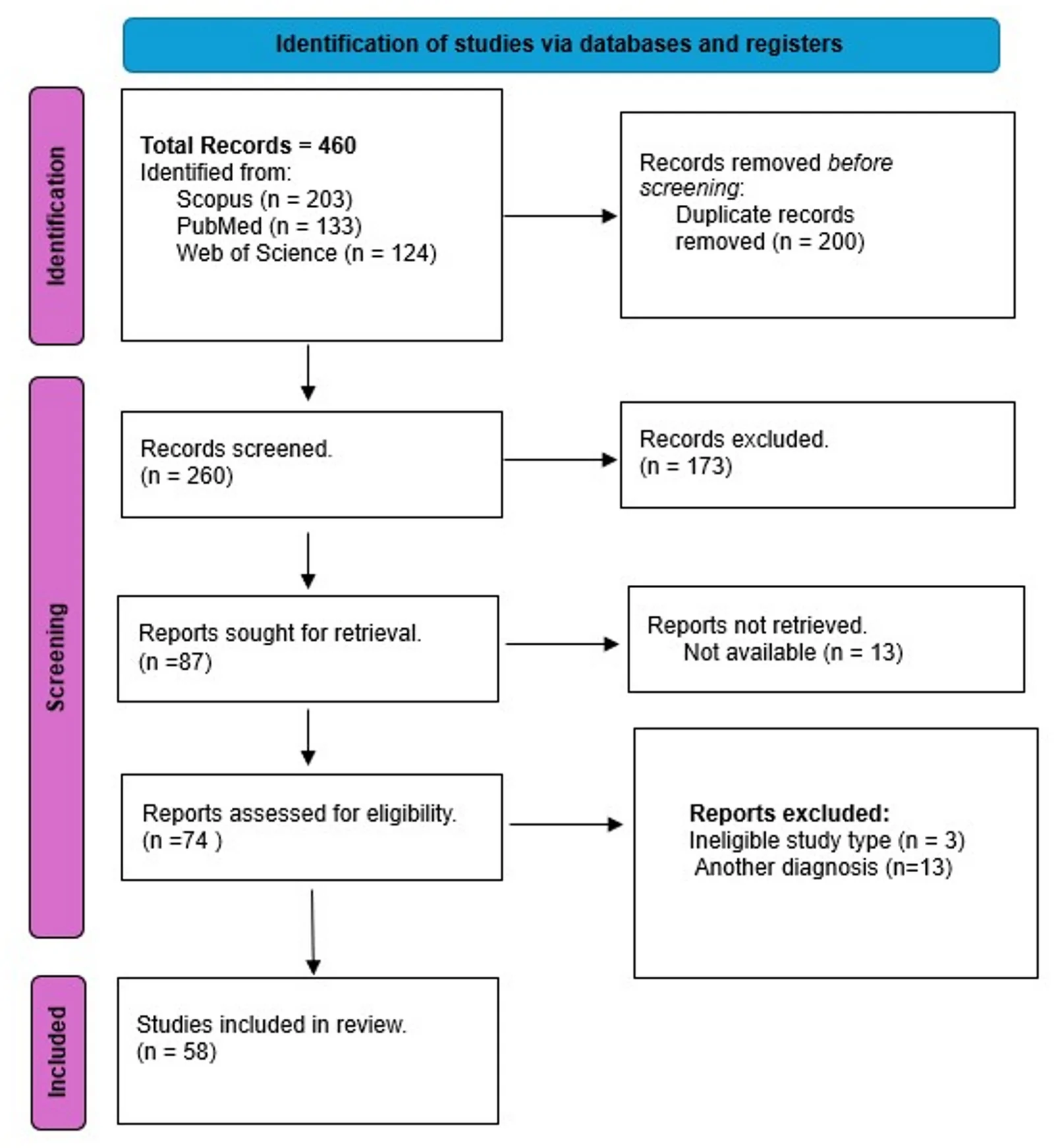
PRISMA flow chart describing the study selection process

An initial search of electronic databases yielded 460 records, which was reduced to 260 following the removal of 200 duplicates. During the primary screening of titles and abstracts, 173 records were excluded for failing to meet the inclusion criteria. The remaining 87 reports were sought for full-text evaluation; however, 13 reports could not be retrieved. A rigorous eligibility assessment was performed on the remaining 74 reports, resulting in the exclusion of 16 studies: three were omitted due to ineligible study designs and 13 were excluded because the patient cohorts presented with diagnoses other than IA. Consequently, 58 studies met the eligibility criteria and were included in the final analysis. The systematic selection process is illustrated in the PRISMA flow diagram (Fig. 1).

### Patient characteristics, clinical background, and temporal and geographic distribution of reported idiopathic aortitis cases

A total of 79 patients with IA were included. The mean age of the cohort was 46 ± 20 years. The cohort showed a slight male predominance, with 38 patients (53%) being male and 34 (47%) female.

Regarding comorbidities, a history of rheumatic disease was present in seven patients (13%). Cardiometabolic conditions were noted, with hypertension reported in 27 patients (51%) and diabetes mellitus in two patients (6.9%). Prior cardiovascular and vascular events included a history of catheterization in nine patients (24%), stenting in six patients (17%), stroke in four patients (9.8%), peripheral arterial disease (PAD) in two patients (5.4%), and myocardial infarction in one patient (2.8%) (Table 1).

**Table 1:** Patient characteristics and clinical background of idiopathic aortitis cases

Characteristic	*N*	*N* = 79
Age*	69	46 ± 20
**Gender**	72	
Female		34 (47%)
Male		38 (53%)
**Hypertension**	53	
No		26 (49%)
Yes		27 (51%)
**DM**	29	2 (6.9%)
**Rheumatic Disease**	56	7 (13%)
**History Cathetarization**	37	9 (24%)
**Stenting**	36	6 (17%)
**Myocardial Infarction**	36	1 (2.8%)
**stroke**	41	4 (9.8%)
**PAD**	37	2 (5.4%)

*Results were reported as mean (standard deviation).N represents the number of available records for each variable.DM, diabetes mellitus; PAD, peripheral arterial disease

**Fig. 2:**
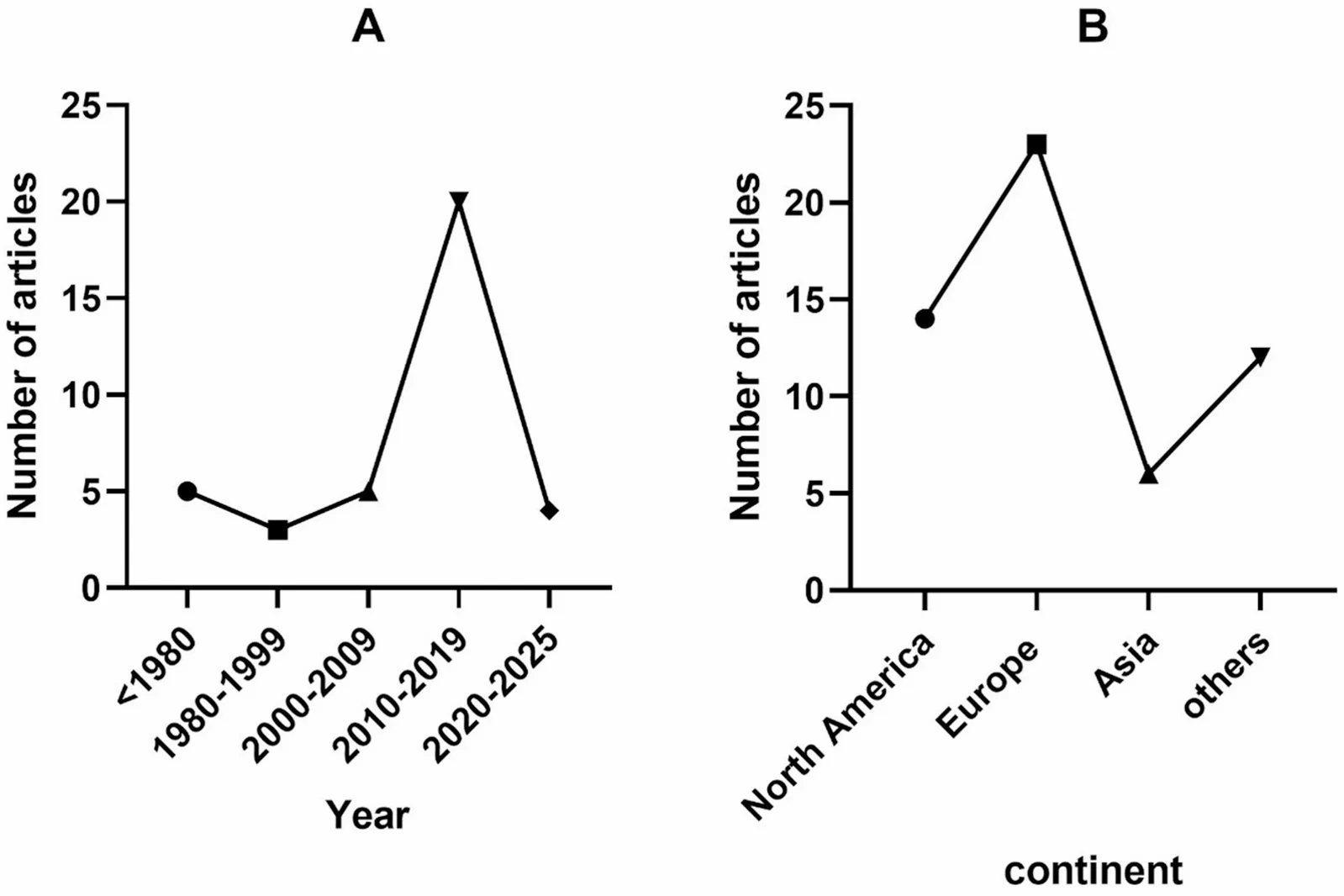
Distribution of published idiopathic aortitis articles by region and year

The temporal distribution of the literature demonstrates that few cases were reported before 1980 (5 articles) and during 1980–1999 (3 articles). A gradual increase was observed in the 2000–2009 period (5 articles), followed by a marked peak between 2010 and 2019 (20 articles). Between 2020 and 2025, 4 cases have been reported. The geographic distribution of cases sorted by continent reveals that the majority of cases originated from Europe (23 articles), followed by North America (14 articles), other regions (12 articles), and Asia (6 articles) (Fig. 2).

### Clinical presentation, complications, and laboratory findings of idiopathic aortitis

Patients with IA most commonly presented with constitutional and pain-related symptoms, with a mean symptom duration of 8 ± 10 months. Weight loss was the most frequently reported symptom, occurring in 26 patients (58%), followed by fatigue in 23 patients (53%), and fever in 24 patients (48%). Abdominal pain was present in 21 patients (51%), while malaise was reported in 16 patients (39%). Musculoskeletal or ischemic symptoms were also noted, including back pain in 13 patients (33%), chest pain in 10 patients (21%), anginal pain in 5 patients (11%), and claudication in 5 patients (11%)

Regarding disease-related complications, aortic regurgitation was the most prevalent, affecting 14 patients (56%), followed by aortic stenosis in 7 patients (35%). Acute aortic events included aortic dissection reported in three patients (11%) and aortic rupture in one patient (3.7%). The disease was misdiagnosed in nine cases (15%). Laboratory evaluation demonstrated evidence of systemic inflammation, with a mean white blood cell (WBC) count of 8,673 ± 4,665. Mean inflammatory markers were elevated, with a C-reactive protein (CRP) of 52 ± 74 and an erythrocyte sedimentation rate (ESR) of 50 ± 37, consistent with active inflammatory disease. Mean hemoglobin (Hb) was 10.82 ± 2.38 (Table 2).

**Table 2:** Clinical presentation and laboratory findings in patients with idiopathic aortitis

Characteristic	*N*	*N* = 79
Duration of Symptoms*	28	8 ± 10
Chest Pain	47	10 (21%)
Anginal pain	44	5 (11%)
Back Pain	40	13 (33%)
Abdominal Pain	41	21 (51%)
Fever	50	24 (48%)
Fatigue	43	23 (53%)
Weight Loss	45	26 (58%)
Malaise	41	16 (39%)
Night sweats	45	4 (8.9%)
claudication	44	5 (11%)
Aortic Rupture	27	1 (3.7%)
Aortic dissection	28	3 (11%)
Aortic regurgitation	25	14 (56%)
Aortic stenosis	20	7 (35%)
Was this Disease Misdiagnosis	61	9 (15%)
WBC	23	8,673 ± 4,665
ESR*	53	50 ± 37
CRP*	33	52 ± 74
Hb*	20	10.82 ± 2.38

*Results were reported as mean (standard deviation).N represents the number of available records for each variable.WBC, white blood cell count; ESR, erythrocyte sedimentation rate; CRP, C-reactive protein; Hb, hemoglobin

### Diagnostic evaluation and management

The diagnostic evaluation of patients with IA involved multiple imaging modalities. Computed tomography (CT) was the most frequently utilized imaging modality, performed in 45 patients (80%), and showed abnormal findings in all 27 reported cases (100%). X-ray imaging was conducted in 50 patients (78%), revealing abnormalities in 19 cases (79%). Ultrasound (US) was performed in 24 patients (44%), with abnormal findings reported in 15 cases (60%). Magnetic resonance imaging (MRI) was performed in nine patients (18%), demonstrating abnormalities in six cases (75%). Biopsy was performed prior to surgery in only two cases (3.1%).

Involvement of the abdominal aorta was most common, occurring in 47 patients (59%), followed by the ascending aorta (29%), descending aorta (28%), aortic arch (25%), and aortic root (19%). The condition was noted as an incidental finding in five cases (7.6%). Regarding management strategies, 27 patients (44%) underwent surgical treatment only, 19 patients (31%) received mixed management (combined medical and surgical), and 15 patients (25%) received medical management only. Corticosteroids were utilized in 24 patients (51%) for a mean duration of 60 ± 71 months, while immunosuppressants other than steroids were used in 10 patients (21%). Emergent presentation occurred in 24 cases (37%), and a total of 12 deaths (20%) were reported (Table 3).

**Table 3:** Imaging findings, management strategies, and outcomes in patients with idiopathic aortitis

Characteristic	*N*	*N* = 79
**Us done**	55	24 (44%)
**Us abnormal findings**	25	15 (60%)
**X-ray done**	64	50 (78%)
**Abnormal finding on chest X-ray**	24	19 (79%)
**CT done**	56	45 (80%)
**Abnormal finding on CT**	27	27 (100%)
**Mri done**	51	9 (18%)
**Abnormal finding on MRI**	8	6 (75%)
**Surveillance**	27	17 (63%)
**Biopsy performed Prior to surgery**	65	2 (3.1%)
**Management**	61	
Medical Only		15 (25%)
Mixed		19 (31%)
Surgical only		27 (44%)
**Abdominal Aorta Involved**	79	47 (59%)
**Ascending Aorta Involved**	79	23 (29%)
**Aortic Arch Involved**	79	20 (25%)
**Aortic Root Involved**	79	15 (19%)
**Descending Aorta Involved**	79	22 (28%)
**Incidental Finding**	66	5 (7.6%)
**Corticosteroids**	47	24 (51%)
Corticosteroids Duration (Months)*	12	60 ± 71
**Hypertensive Medications**	44	22 (50%)
**Beta Blockers**	34	5 (15%)
**Calcium Channel Blockers**	33	3 (9.1%)
**Diuretics**	35	8 (23%)
**Immunosuppressants other Than steroids**	48	10 (21%)
**Corticosteroids prior to surgery**	29	3 (10%)
**Emergent presentation**	65	24 (37%)
**Valvular complications**	65	16 (25%)
**Electro-cardiac complications**	68	11 (16%)
**Low Ejection Fraction**	25	7 (28%)
**Recurrence**	49	5 (10%)
**Death**	61	12 (20%)

*Results were reported as mean (standard deviation).N represents the number of available records for each variable.US, ultrasound; CT, computed tomography; MRI, magnetic resonance imaging. “Mixed” management refers to combined medical and surgical treatment

### Mortality-based differences in idiopathic aortitis patients

When comparing characteristics based on mortality status, patients who died had a significantly lower mean age compared to those who survived (18 ± 16 vs. 48 ± 17, *P* < 0.001). There were no statistically significant differences in mortality based on gender (*P* = 0.813), hypertension (*P* = 0.089), or the presence of various symptoms such as chest pain, fever, fatigue, or weight loss. Similarly, laboratory values including WBC, ESR, and Hb did not significantly differ between the two groups.

However, imaging and anatomical involvement showed notable differences. Diagnostic imaging, including US (*P* = 0.001) and CT (*P* < 0.001), was performed significantly more often in patients who survived compared to those who died. Furthermore, involvement of the descending aorta was significantly more common among patients who died (75% vs. 27%, *P* = 0.005). Additionally, patients who survived were significantly more likely to have received corticosteroids (63% vs. 0%, *P* = 0.002) and to have undergone surveillance (81% vs. 0%, *P* < 0.001). No significant differences were observed in management strategies (medical, mixed, or surgical) between the survival groups (*P* = 0.933) (Table 4).

**Table 4:** Comparison of clinical characteristics, imaging, management, and outcomes by mortality status in idiopathic aortitis

Variable	Alive	Dead	*P*-value
**Age**	48 ± 17	18 ± 16	**< 0.001**
**Gender**			0.813
Female	22 (49%)	6 (55%)	
Male	23 (51%)	5 (45%)	
**HTN**	16 (62%)	9 (100%)	0.089
**DM**	2 (17%)	0 (0%)	> 0.999
**Rheumatic disease**	6 (19%)	0 (0%)	0.585
**History of catheterisation**	8 (42%)	1 (50%)	> 0.999
**stenting**	6 (32%)	0 (0%)	> 0.999
**stroke**	3 (13%)	1 (50%)	0.717
**Duration of Symptoms**	7 ± 10	10 ± 10	0.678
**Chest Pain**	10 (33%)	0 (0%)	> 0.999
**Anginal**	5 (19%)	0 (0%)	> 0.999
**Back pain**	9 (39%)	0 (0%)	> 0.999
**Abdominal pain**	10 (42%)	0 (0%)	> 0.999
**Fever**	11 (34%)	1 (50%)	> 0.999
**fatigue**	11 (42%)	0 (0%)	> 0.999
**Weight loss**	14 (50%)	0 (0%)	> 0.999
**malaise**	4 (17%)	0 (0%)	> 0.999
**Night sweats**	4 (14%)	0 (0%)	> 0.999
**claudication**	3 (12%)	0 (0%)	> 0.999
**Rupture**	0 (0%)	1 (14%)	0.576
**Aortic dissection**	2 (9.5%)	1 (14%)	> 0.999
**Aortic regurgitation**	14 (58%)	0 (0%)	0.902
**Aortic stenosis**	6 (33%)	1 (50%)	> 0.999
**Disease misdiagnosis**	7 (19%)	2 (22%)	> 0.999
**WBC**	7,837 ± 4,057	14,667 ± 5,974	0.179
**ESR**	44 ± 35	24 ± 23	0.181
**Hb**	11.35 ± 2.19	9.88 ± 2.78	0.381
**US done**	24 (75%)	0 (0%)	**0.001**
**X-ray done**	23 (66%)	9 (82%)	0.524
**Abnormal finding on xray**	12 (71%)	7 (100%)	0.289
**CT done**	28 (88%)	1 (13%)	**< 0.001**
**Abnormal finding on CT**	26 (100%)	1 (100%)	> 0.999
**MRI done**	9 (32%)	0 (0%)	0.209
**Surveillance**	17 (81%)	0 (0%)	**< 0.001**
**Biopsy performed prior to surgery**	1 (2.5%)	0 (0%)	> 0.999
**Management**			0.933
Medical Only	12 (31%)	3 (38%)	
Mixed	16 (41%)	3 (38%)	
Surgical only	11 (28%)	2 (25%)	
**Abdominal Aorta Involved**	25 (51%)	5 (42%)	0.796
**Ascending Aorta Involved**	20 (41%)	3 (25%)	0.496
**Aortic Arch Involved**	13 (27%)	7 (58%)	0.078
**Aortic Root Involved**	13 (27%)	2 (17%)	0.736
**Descending Aorta Involved**	13 (27%)	9 (75%)	**0.005**
**Incidental finding**	5 (12%)	0 (0%)	0.624
**Corticosteroids**	24 (63%)	0 (0%)	**0.002**
**Hypertensive Medications**	18 (69%)	1 (50%)	> 0.999
**Beta Blockers**	5 (31%)	0 (0%)	0.926
**Calcium Channel Blockers**	1 (6.7%)	0 (0%)	> 0.999
**Diuretics**	6 (35%)	0 (0%)	0.832
**Immunosupressants other Than steroids**	10 (26%)	0 (0%)	0.211
**Corticosteroids prior to surgery**	3 (11%)	0 (0%)	> 0.999
**Emergent presentation**	15 (38%)	6 (67%)	0.221
**Valvular complications**	14 (35%)	2 (22%)	0.730
**Electro-cardiac complications**	7 (17%)	4 (36%)	0.329
**Low Ejection Fraction**	6 (35%)	1 (13%)	0.480

HTN, hypertension; DM, diabetes mellitus; WBC, white blood cell count; ESR, erythrocyte sedimentation rate; Hb, hemoglobin; US, ultrasound; CT, computed tomography; MRI, magnetic resonance imaging.Surveillance is defined as longitudinal clinical and imaging follow-up, including serial assessments for disease progression, recurrence, and development of aortic complications rather than providing medical or surgical intervention

## Discussion

IA remains a rare, poorly understood condition, which is defined clinically, with laboratorial and histopathological characteristics helping its diagnosis. This systematic review and meta-analysis of the published reports provide a better understanding of the patients’ population and the disease characteristics. In addition, it provided an in-depth analysis of the histopathological findings and the gender-based differences among IA patients.

Even though the etiology of IA is unknown [12], its sex distribution in our cohort differed from that of other major causes of aortitis, namely giant cell arteritis and Takayasu arteritis, which predominantly affect females [1, 2], In contrast, IA in our study demonstrated an approximately equal female-to-male ratio. Despite autoimmune disease being considered a risk factor for IA [13], and in addition to the balanced sex distribution, concurrent autoimmune conditions were identified in 12.5% of our cohort. This prevalence is slightly higher than the estimated 5–10% prevalence of autoimmune diseases in the general population [14, 15], which shows a slightly higher prevalence of autoimmune conditions among IA patients. Additionally, when compared to its autoimmune differentials, IA mean age at diagnosis, as identified in our study was 52.2 years, when compared to Takayasu arteritis, which mainly affects much younger female patients with a mean age of 25 years [16]. On the other hand, IA had a slightly younger age of presentation compared to giant cell arteritis, which have an average age of presentation of 74 to 76 years, and its incidence peaks at 80 years [17, 18]. Another noteworthy observation of our study population, that the prevalence of other cardiovascular conditions and cardiovascular risk factors was comparable to general population [19], making it one of the differentiating factors between other cardiovascular conditions.

Previous studies have shown that male-gender by itself, is associated with worse outcomes in patients with non-infectious aortitis, including cases of IA [20], our study, showed that the male population doesn’t have significantly higher mortality rate.

Management of IA usually involves corticosteroids, which are considered the first line management, often with other immunosuppressive medications [21]. In our analysis, 51% of the patients received corticosteroids, and 21% of them received immunosuppressants other than corticosteroids. In addition, 44% received only surgical intervention without any other medical intervention. These surgical interventions are required when there is a form of aneurysm, dissection, or other vascular complications [22, 23]. In our cohort, 3.7% had a ruptured aorta and dissection in 11% on presentation.

Outcomes are generally worse than other forms of aortitis in IA patients, with high rates of vascular complications and mortality among patients [24]. The mortality rate was 20% among or patients populations of IA. In addition, the recurrence rate was 10% among patients. Younger age group was a significant predictor of worse outcomes among IA patients, which is similar to the findings in previous articles [24, 25]. This difference may be explained by histopathological variations between younger and older patients. In older patients, inflammation predominantly involves the intima and inner media, accompanied by prominent intimal thickening and a relatively spared, less fibrotic adventitia. In contrast, younger patients typically demonstrate inflammation concentrated within the outer media and adventitia, along with marked, often paucicellular adventitial fibrous expansion that exceeds that observed in other forms of aortitis [22, 26]. Similar outcomes across treatment strategies in idiopathic aortitis can be explained by the fact that it is localized, histologically-discovered process rather than an active systemic vasculitis, so the therapeutic “lever” that medical therapy would pull is frequently small. In addition, our study found that involvement of the descending aorta was a predictor for mortality among patients. On the other hand, the use of corticosteroids was only present among patients who were alive. This might be due to the more severe, life-threatening presentations of the disease.

Our study which was based exclusively on published case reports and series of IA patients, has several important limitations including the fact that case reports showed significant heterogeneity and inconsistency in data reporting. This restricted the ability to perform quantitative data pooling for many key variables. In addition, due to the retrospective, observational nature of case reports, systematic long-term follow-up was generally unavailable. This prevented reliable assessment of long-term outcomes, disease progression, or diagnostic evolution over time, and precluded meaningful head-to-head comparisons with similar differential diagnoses. These limitations highlight the challenges in synthesizing evidence for this rare condition and underscore the need for prospective, multicenter registries with standardized reporting to better understand its natural history, management, and prognosis. Another limitation of this study is the inconsistent reporting of aneurysmal aortic disease across the included studies. While a small number of studies explicitly reported its presence or absence, most did not provide this information, precluding accurate assessment of its prevalence and limiting our ability to evaluate its potential influence on treatment strategies and clinical outcomes.

## Conclusion

In conclusion, this systematic review and meta-analysis delineates the clinical profile, management strategies, and survival outcomes of IA based on the available published cases, highlighting it as a rare entity distinct from other large-vessel vasculitides such as giant cell arteritis and Takayasu arteritis. Our findings demonstrate a balanced sex distribution and frequent presentation with constitutional symptoms and significant systemic inflammation, underscoring IA’s complex clinical picture. While diagnostic imaging, particularly CT, is universally utilized and highly sensitive, the management of IA remains heterogeneous, spanning medical, surgical, and mixed approaches. Crucially, our mortality analysis reveals that a younger age at presentation and involvement of the descending aorta are significant predictors of mortality, whereas the administration of corticosteroids and clinical surveillance are strongly associated with survival. Despite the limitations of our study, including reliance on retrospective case reports and variable follow-up durations, these insights advance our understanding of IA’s prognostic factors and treatment modalities. Future research should prioritize prospective, multicenter registries with standardized protocols to investigate its etiology, establish evidence-based management guidelines, and further clarify long-term prognostic factors, ultimately improving patient outcomes in this severe and understudied condition.

## Data Availability

No datasets were generated or analysed during the current study.

## Abbreviations

ANA: Antinuclear Antibody
CRP: C-reactive Protein
CT: Computed Tomography
ESR: Erythrocyte Sedimentation Rate
GCA: Giant Cell Arteritis
Hb: Hemoglobin
IA: Idiopathic Aortitis
IgG4: Immunoglobulin G4
MRA: Magnetic Resonance Angiography
MRI: Magnetic Resonance Imaging
PAD: Peripheral Arterial Disease
PET-CT: Positron Emission Tomography – Computed Tomography
PRISMA: Preferred Reporting Items for Systematic Reviews and Meta-analysis
SD: Standard Deviation
TAK: Takayasu Arteritis
US: Ultrasound
WBC: White Blood Cell

## References

[1] Gornik HL, Creager MA (2008) Aortitis Circulation 117:3039–3051. 10.1161/CIRCULATIONAHA.107.760686 PMC2759760 18541754

[2] Rojo-Leyva F, Ratliff NB, Cosgrove DM, Hoffman GS (2000) Study of 52 patients with idiopathic aortitis from a cohort of 1,204 surgical cases. Arthritis Rheum 43:901–907. 10.1002/1529-0131(200004)43:4%3C901::AID-ANR23%3E3.0.CO;2-U 10765937

[3] Jennette JC, Falk RJ, Bacon PA et al (2013) 2012 revised International Chapel Hill Consensus Conference Nomenclature of Vasculitides. Arthritis Rheum 65:1–11. 10.1002/art.37715 23045170

[4] Pipitone N, Salvarani C (2011) Idiopathic aortitis: an underrecognized vasculitis. Arthritis Res Ther 13:119. 10.1186/ar3389 PMC3239344 21787438

[5] Miller DV, Isotalo PA, Weyand CM et al (2006) Surgical pathology of noninfectious ascending aortitis: A study of 45 cases with emphasis on an isolated variant. Am J Surg Pathol 30:1150–1158. 10.1097/01.pas.0000213293.04026.ec 16931960

[6] Chatterjee S, Flamm SD, Tan CD, Rodriguez ER (2014) Clinical diagnosis and management of large vessel vasculitis: Takayasu arteritis. Curr Cardiol Rep 16:499. 10.1007/s11886-014-0499-y 24893936

[7] Mukhtyar C, Guillevin L, Cid MC et al (2009) EULAR recommendations for the management of large vessel vasculitis. Ann Rheum Dis 68:318–323. 10.1136/ard.2008.088351 18413441

[8] Macedo TA, Stanson AW, Oderich GS et al (2004) Infected aortic aneurysms: imaging findings. Radiology 231:250–257. 10.1148/radiol.2311021700 15068950

[9] Pugh D, Grayson P, Basu N, Dhaun N (2021) Aortitis: recent advances, current concepts and future possibilities. Heart 107:1620–1629. 10.1136/heartjnl-2020-318085 33593995

[10] Page MJ, McKenzie JE, Bossuyt PM et al (2021) The PRISMA 2020 statement: an updated guideline for reporting systematic reviews. 10.1136/bmj.n71 PMC8008539 33781348

[11] Ouzzani M, Hammady H, Fedorowicz Z, Elmagarmid A (2016) Rayyan—a web and mobile app for systematic reviews. Syst Rev 5:210. 10.1186/s13643-016-0384-4 PMC5139140 27919275

[12] Espitia O, Bruneval P, Assaraf M et al (2023) Long-Term Outcome and Prognosis of Noninfectious Thoracic Aortitis. J Am Coll Cardiol 82:1053–1064. 10.1016/j.jacc.2023.06.031 37673506

[13] Schmidt J, Sunesen K, Kornum JB et al (2011) Predictors for pathologically confirmed aortitis after resection of the ascending aorta: a 12-year Danish nationwide population-based cross-sectional study. Arthritis Res Ther 13:R87. 10.1186/ar3360 PMC3218902 21676237

[14] Cooper GS, Bynum MLK, Somers EC (2009) Recent Insights in the Epidemiology of Autoimmune Diseases: Improved Prevalence Estimates and Understanding of Clustering of Diseases. J Autoimmun 33:197–207. 10.1016/j.jaut.2009.09.008 PMC2783422 19819109

[15] Shapira Y, Agmon-Levin N, Shoenfeld Y (2010) Defining and analyzing geoepidemiology and human autoimmunity. J Autoimmun 34:J168–J177. 10.1016/j.jaut.2009.11.018 20034761

[16] Alnabwani D, Patel P, Kata P et al (2021) The epidemiology and clinical manifestations of takayasu arteritis: a descriptive study of case reports. Cureus 13:e17998. 10.7759/cureus.17998 PMC8519497 34667674

[17] Salvarani C, Cantini F, Boiardi L, Hunder GG (2002) Polymyalgia rheumatica and giant-cell arteritis. N Engl J Med 347:261–271. 10.1056/NEJMra011913 12140303

[18] Hoffman GS (2016) Giant Cell Arteritis. Ann Intern Med 165:ITC65–ITC80. 10.7326/AITC201611010 27802475

[19] Aggarwal R, Yeh RW, Joynt Maddox KE, Wadhera RK (2023) Cardiovascular Risk Factor Prevalence, Treatment, and Control in US Adults Aged 20 to 44 Years, 2009 to March 2020. JAMA 329:899–909. 10.1001/jama.2023.2307 PMC9986841 36871237

[20] Ferfar Y, Morinet S, Espitia O et al (2021) Spectrum and Outcome of Noninfectious Aortitis. J Rheumatol 48:1583–1588. 10.3899/jrheum.201274 34210830

[21] Ahmad N, Andev R, Verdiyeva A, Dubey S (2023) Single centre experience of 120 patients with non-infectious aortitis: Clinical features, treatment and complications. Autoimmun Rev 22:103354. 10.1016/j.autrev.2023.103354 37142195

[22] Cacoub P, Vieira M, Langford CA et al (2025) Large-vessel vasculitis. Lancet 406:2017–2032. 10.1016/S0140-6736(25)01436-9 40939604

[23] Shchetynska-Marinova T, Amendt K, Sadick M et al (2021) Aortitis - An Interdisciplinary Challenge. Vivo 35:41–52. 10.21873/invivo.12230 PMC7880770 33402448

[24] Espitia O, Samson M, Le Gallou T et al (2016) Comparison of idiopathic (isolated) aortitis and giant cell arteritis-related aortitis. A French retrospective multicenter study of 117 patients. Autoimmun Rev 15:571–576. 10.1016/j.autrev.2016.02.016 26903476

[25] Czerny M, Grabenwöger M, Berger T et al (2024) EACTS/STS Guidelines for diagnosing and treating acute and chronic syndromes of the aortic organ. Eur J Cardiothorac Surg 65:ezad426. 10.1093/ejcts/ezad426 38408364

[26] Sandovici M, van der Geest KSM, Reitsema RD et al (2026) Challenges and Future Trends in Large Vessel Vasculitis. Circulation 153:2064–2083. 10.1161/CIRCULATIONAHA.125.077491 PMC13288755 42330100

